# Microglia-Mediated Neuroinflammation and Neurotrophic Factor-Induced Protection in the MPTP Mouse Model of Parkinson’s Disease-Lessons from Transgenic Mice

**DOI:** 10.3390/ijms17020151

**Published:** 2016-01-25

**Authors:** Venissa Machado, Tanja Zöller, Abdelraheim Attaai, Björn Spittau

**Affiliations:** 1 Institute for Anatomy and Cell Biology, Department of Molecular Embryology, Albert-Ludwigs-University Freiburg, Albertstraße 17, Freiburg 79104, Germany; venissa.machado@sgbm.uni-freiburg.de (V.M.); tanja.zoeller@anat.uni-freiburg.de (T.Z.); abdelraheim.attaai@anat.uni-freiburg.de (A.A.); 2 Spemann Graduate School of Biology and Medicine (SGBM), Albert-Ludwigs-University Freiburg, Germany, Albertstraße 19A, Freiburg 79104, Germany; 3 Faculty of Biology, Albert-Ludwigs-University Freiburg, Schänzlestraße 1, Freiburg 79104, Germany; 4 Institute for Anatomy and Cell Biology, Department of Neuroanatomy, Albert-Ludwigs-University Freiburg, Albertstraße 17, Freiburg 79104, Germany; tanja.zoeller@anat.uni-freiburg.de; 5 Department of Anatomy and Histology, Faculty of Veterinary Medicine, Assiut University, Assiut 71526, Egypt

**Keywords:** MPTP, neuroinflammation, microglia, Parkinson’s disease, neurotrophic factors, neuroprotection

## Abstract

Parkinson’s disease (PD) is a neurodegenerative disease characterised by histopathological and biochemical manifestations such as loss of midbrain dopaminergic (DA) neurons and decrease in dopamine levels accompanied by a concomitant neuroinflammatory response in the affected brain regions. Over the past decades, the use of toxin-based animal models has been crucial to elucidate disease pathophysiology, and to develop therapeutic approaches aimed to alleviate its motor symptoms. Analyses of transgenic mice deficient for cytokines, chemokine as well as neurotrophic factors and their respective receptors in the 1-methyl-4-phenyl-1,2,3,6-tetrahydropyridine (MPTP) model of PD have broadened the current knowledge of neuroinflammation and neurotrophic support. Here, we provide a comprehensive review that summarises the contribution of microglia-mediated neuroinflammation in MPTP-induced neurodegeneration. Moreover, we highlight the contribution of neurotrophic factors as endogenous and/or exogenous molecules to slow the progression of midbrain dopaminergic (mDA) neurons and further discuss the potential of combined therapeutic approaches employing neuroinflammation modifying agents and neurotrophic factors.

## 1. Introduction

Parkinson’s disease (PD) is the second most common neurodegenerative disease after Alzheimer’s disease (AD) and affects 1%–2% of the population over 60 years of age [1,2]. PD is characterised by a progressive loss of dopaminergic (mDA) neurons in the substantia nigra (SN) and a subsequent decrease in dopamine levels in the basal ganglia (CPu) [3]. This reduction in dopamine is responsible for the typical clinical symptoms of akinesia/bradykinesia, in combination with resting tremor, rigidity and gait disturbances. However, several non-motor clinical symptoms such as autonomic dysfunctions, anxiety, depression, cognitive impairments as well as impaired olfaction have been associated with PD and are not necessarily related to the lack of dopamine [4,5,6]. The histopathological hallmarks of PD are reduced numbers of mDA neurons and the presence of intracellular protein aggregate inclusions called Lewy bodies [7]. α-synuclein, the main component of Lewy bodies [8], is thought to provoke intracellular stress responses in neurons eventually resulting in the degeneration of Lewy body-containing neurons. Interestingly, the appearance of Lewy bodies is not restricted to mDA neurons and has been reported in other brain areas depending on disease severity. These observations led to the Braak hypothesis, which defines PD as a neurodegenerative diseases that ascends from the dorsal motor nucleus of the vagus nerve, to lower brainstem areas including the ventral midbrain, and finally to neocortical regions [9]. However, the presence of Lewy bodies alone does not explain the onset and progression of PD [10], indicating that a secondary effect triggered by neuronal impairment might be responsible for its progressive nature. Neuroinflammatory reactions have been described in virtually all PD cases, with several studies supporting the hypothesis that microglia-mediated neuroinflammatory responses exacerbate the loss of mDA neurons and, thus, worsen the clinical symptoms [11,12].

In order to understand the pathomechanisms underlying PD and to recapitulate the contribution of microglia-driven inflammatory responses, toxin-based mouse models have extensively been used in the past decades [13]. The most commonly used toxins to induce degeneration of mDA neurons are 6-hydroxydopamine (6-OHDA) and 1-methyl-4-phenyl-1,2,3,6-tetrahydropyridine (MPTP), both of which are relatively selective for catecholaminergic neurons [14].

In this comprehensive review, we summarise the neuroinflammatory responses in the MPTP mouse model of PD, and highlight the contribution of neuroinflammation from a collection of studies where the outcomes of transgenic mice for cytokines/chemokines and their respective receptors have been addressed after MPTP intoxication. We further review the role of neurotrophic factors as endogenous cues to inhibit MPTP-induced neurodegeneration, and propose that future treatment strategies for PD might include a combination of neurotrophic support and molecules targeting microglia-mediated neuroinflammation.

MPTP is an analogue of the narcotic meperidine, and was accidentally discovered when drug addicts developed a Parkinsonian syndrome after intravenous injections of MPPP (1-methyl-4-phenyl-propion-oxypiperidine) with a full spectrum of clinical motor symptoms [15]. The neurotoxic effects caused by MPTP had similar clinical and neuropathological findings as those observed in PD patients, with the exception of Lewy bodies. These observations led to the establishment of MPTP as a common neurotoxin to induce Parkinsonism in several animal models [16]. When administered systemically (either as intraperitoneal or intravenous injections), MPTP easily crosses the blood-brain-barrier (BBB) owing to its lipophilic nature. Astrocytic monoamine oxidase B further converts MPTP to 1-methyl-4-phenyl-2,3-dihydropyridium (MPDP), which spontaneously oxidises to yield the polar molecule 1-methyl-4-phenylpyridinium (MPP^+^). MPP^+^ is then released into the extracellular space, but due its polar nature, cannot freely enter cells [17,18,19]. Th-ir (Tyrosine hydroxylase-immunoreactive) mDA neurons and other catecholaminergic neurons are especially sensitive to MPTP-induced degeneration due to the selective uptake of MPP^+^ by the dopamine transporter (DAT) and noradrenaline and serotonin transporters [20,21,22]. After uptake into mDA neurons, MPP^+^ binds to the vesicular monoamine transporter (VMAT) and further accumulates in the mitochondria as well as in the cytoplasm where it can interact with several enzymes [23,24]. The neurotoxic effects of MPTP are caused by an inhibition of the complex I of the electron transport chain, leading to increased reactive oxygen species production and oxidative stress [25,26,27].

The selective degeneration of mDA neurons by MPTP makes it one of the most commonly used toxins to induce PD in rodents. However, as rats are resistant to its effects [28,29], mice are routinely used as model organisms in this toxin paradigm. Interestingly, the inbred strain C57BL/6 has been shown to be more susceptible to MPTP than the BALB/c (BALB) strain [30,31], and is therefore used in the majority of studies to date. According to the study question, different MPTP application regimes can be used and are summarised below according to Schmidt and Ferger [32] and Schober [14]:

1. Pre-symptomatic PD model: acute single MPTP application at low dose (1 × 10–20 mg/kg).
2. Immediate onset PD model [33]: acute repetitive MPTP application at intermediate doses (4 × 20 mg/kg at 2 h intervals).
3. Sub-chronic PD model [34,35]: MPTP injections (1–2) at doses about 20–30 mg/kg for up to 5 consecutive days.
4. Progressive chronic PD model [36]: daily MPTP injections at low doses (4 mg/kg) over 20 days.

It is worth mentioning that the sub-chronic and chronic models are characterised by degeneration of mDA neurons and, thus, most likely resemble the situation in human PD cases. Apart from these four treatment paradigms, several studies have used slightly modified protocols to address questions of neurodegeneration and neuroinflammation [32].

Although MPTP is one of the most potent toxins used to induce specific degeneration of mDA neurons, it has to be noted that MPTP is a hazardous and dangerous agent and users have to follow strict rules concerning storage, handling and disposal of the neurotoxin [16].

## 2. Neuroinflammatory Responses in the 1-Methyl-4-phenyl-1,2,3,6-tetrahydropyridine (MPTP) Mouse Model for Parkinson’s Disease

### 2.1. Temporal and Spatial Glia Reactions after MPTP Intoxication

Systemic injections of MPTP result in a rapid onset of neuroinflammatory responses in the SN as well as in the CPu. These astroglia- and microglia-mediated responses are triggered by the impairment of mDA neuron function and maintenance, making the MPTP model suitable to analyse the associated neuroinflammatory changes [37]. Due to its lipophilic nature and the ability to cross the BBB, the MPTP model has the advantage that microglia are not activated by direct injections or by mechanical manipulations of the central nervous system (CNS). This makes it superior to the 6-OHDA model, wherein the toxin has to be injected directly into the CNS parenchyma [14]. Microglial reactions have been simply categorised into M1 and M2 reactions, which in theory, resemble two opposite activation states. While M1 activation is supposed to promote acute neuroinflammation, cellular and/or neurotoxicity, M2 activation is believed to mediate anti-inflammatory effects associated with tissue regeneration and wound healing. However, these theoretical M1/M2 activation states are the end-points of a microglia activation continuum. The influence of these activation states in neurodegenerative diseases are yet only partially understood, and need to be further addressed [38].

The first studies addressing microglial reactions in the MPTP mouse model demonstrated a distinct microglia activation characterised by an increase in cell numbers and typical changes in microglia morphology, such as increased lectin staining, larger cell bodies, reduced ramification and thickening of cellular processes [39,40]. These microglial reactions could be observed in the SN and in the CPu during the first two weeks after MPTP intoxication. Moreover, signs of active phagocytosis and morphological features indicating secretion of biologically active substances could be detected at the ultrastructural level. Kohutnicka and colleagues further provided evidence for a delayed astrocytic response, which might also be involved in mediating the neuroinflammatory response [39]. These results suggested that microglial activation, which preceded neuron degeneration, might be involved in the progression of mDA neuron damage after MPTP intoxication. Interestingly, inhibition of microglial activation by application of minocycline, a tetracycline derivative, mitigated both the decrease of nigrostriatal mDA neurons and the formation of nitro-tyrosine produced by MPTP. Furthermore, minocycline not only prevented MPTP-induced microglial activation but also inhibited the formation of mature Il-1β, activation of NADPH-oxidase, and inducible nitric oxide synthase (iNOS), all of which are known microglial-derived cytotoxic mediators [41].

Detailed analyses of the spatiotemporal expression of inflammatory markers indicated that *Il-1β* expression rapidly increased after MPTP intoxication and peaked at around 6 h [42]. Increases in Tumour necrosis factor α (*Tnf-α*), iNos and Interferon-γ (*Ifn-γ*) were described between 6 h and 24 h after MPTP administration, whereas *Il-10* expression revealed two peaks of induction; 6 h and 3 days after MPTP injection. *Il-6* showed a delayed upregulation at 7 days post MPTP administration. This expression data is in congruence with other studies, thus corroborating the involvement of Tnf-α, iNos, Il-1β and Ifn-γ as key neuroinflammatory players after MPTP intoxication [43,44]. Moreover, the expression levels of receptors *Il-1r1*, *Tnf-αr1* and *Il-6rα* were significantly increased in the SN of MPTP-treated mice, whereas no significant differences were observed in the CPu. Yasuda and colleagues [45] reported that MPTP-sensitive C57BL/6 mice, characteristic for their propensity towards M1 microglia reactions, displayed an increase in the inflammatory markers Il-10, Il-12 p40, Ifn-γ and Mcp-1/Ccl2 (monocyte/macrophage chemoattractant protein) in the cerebrospinal fluid. This increase in expression could not be detected in the MPTP-resilient BALB mice, indicating that the reported susceptibility of C57BL/6 mice for MPTP-induced mDA neurodegeneration [30] might be caused by the prominent neuroinflammatory response of C57BL/6 mice.

An increase in expression of *Mcp-1/Ccl2* after MPTP injections has also been reported [46]. *Mcp-1/Ccl2* was one of the first upregulated inflammatory proteins after MPTP administration, suggesting a role for Mcp-1/Ccl2 as a key component in the neuroinflammatory cascade. Although most studies addressed the transient effects of MPTP injections on microglial reactions, long-term effects could be observed even after 6 months of toxin administration. Microglia in the SN and the CPu of MPTP-treated C57BL/6 mice showed hypertrophic cell bodies as compared to control microglia, underlining that MPTP administration results in the presence of persistent activated microglia [47]. Activation of microglia in the MPTP model does not only trigger a neuroinflammatory cascade characterised by release of cytokines and chemokines, but further results in microglia-neuron interactions, referred to as gliapes, which precede mDA neurodegeneration. A study by Barcia and colleagues [48] demonstrated that activated microglia engulf mDA neurons in a Rock/Cdc42-dependent manner, thereby directly contributing to the decrease in mDA neurons after MPTP administration.

Apart from microglial and astroglial reactions, the latter of which is less well studied and described, peripheral immune cells also play an important role during the course of neuroinflammation in the mouse MPTP model, with reports of it infiltrating CD4^+^ and CD8^+^ T-lymphocytes in the SN [49]. Moreover, these activated lymphocytes showed increased Lymphocyte function-associated antigen 1 (Lfa-1) and CD44 expression. Interestingly, treatment with dexamethasone, an inhibitor of T-cell infiltration and activation, reduced microglia and astroglia reactions and further ameliorated mDA neurodegeneration in the MPTP mouse model. The evidence that degeneration of mDA neurons might be regulated by the adaptive immune system was further supported by a study employing two different immunodeficient mice strains. Using Rag1^−/−^ and Tcrb^−/−^ mice, the authors demonstrated that MPTP-induced mDA neuron degeneration was significantly reduced in the absence of T-cells [50]. However, T-cells represent a heterogeneous population of adaptive immune cells, with regulatory T-cells being primarily described as immunosuppressive, maintaining immune homeostasis and tolerance. Adoptive transfer of immunosuppressive CD4^+^/CD25^+^ regulatory T-cells was able to efficiently protect mDA neurons by T-cell-mediated inhibition of microglia-driven neuroinflammation [51]. Together, these data clearly demonstrate that neuroinflammation is a hallmark of the MPTP mouse model for PD. A central question is whether microglia-mediated neuroinflammation is mechanistically involved in the progression of mDA neurodegeneration, and thus, studies using transgenic mice have been performed to address this question.

### 2.2. Cytokine Signalling

Cytokines are small-secreted proteins, acting as important signalling mediators under homeostatic conditions and during inflammation. Based on their structural homology and function, they are classified into chemokines, interferons, interleukins, lymphokines and tumour necrosis factor [52,53]. Chemokines attract cells to the site of infection and can thus induce gliosis in the CNS.

The innate immune responses mediated by interferons provide a first line of defence against inflammation, and play vital roles in immune surveillance [54,55].

*Ifn-γ* deletion attenuates loss of mDA neurons, their projecting fibres and dopamine levels post MPTP-intoxication [56]. Microglial activation is completely abolished in these *Ifn-γ*^−/−^ mice. Moreover, *Ifn-γ*^−/−^ microglia do not show an increase in size, but do maintain lesion-induced branching behaviour after MPTP treatment. Similarly, *Ifn-γ*^−/−^ astrocytes failed to expand post-MPTP lesion but still exhibited their characteristic size change, indicating that Ifn-γ is not essential for these morphological changes [57].

Deletion of *Cx3cl1* (fractalkine) and its receptor *Cx3cr1*, leads to a dysregulation of microglial reaction and an aggravated response to MPTP [58]. Cardona and colleagues [59] reported a higher loss of Th- and Nissl-positive cells both in *Cx3cl1*^−/−^ and *Cx3cr1*^−/−^ mice. Moreover, in comparison to control microglia, *Cx3cr1*^−/−^ microglia exhibited severe morphological changes in the SN.

In a study assessing the temporal profile of inflammatory mediators, Pattarini and colleagues observed elevated mRNA expression of monocyte chemoattractant protein 1/CC chemokine ligand 2 (*Mcp-1/Ccl2*) immediately after MPTP administration [46]. Interestingly, *Mcp-1/Ccl2*^−/−^ mice had significantly lower levels of macrophage inflammatory protein-1 α/CC chemokine ligand 3 (Mip-1α/Ccl3) and Mip-1β/Ccl4, suggesting its role in MPTP-mediated neuroinflammation. However, mDA neuron losses in *Mcp-1/Ccl2*^−/−^ and WT mice were not significantly different. Of note, this was the only study with our search criteria in which female mice were used for the MPTP model.

Analysis of MPTP-lesioned *Ccl2*^−/−^ (Cysteine–cysteine chemokine ligand-2), *Ccr2*^−/−^ (CC chemokine receptor-2) and double *Ccl2*^−/−^/*Ccr2*^−/−^ mice did not reveal differences in striatal dopamine levels as compared to WT mice, suggesting that the lack of *Ccl2* and *Ccr2* does not protect against MPTP-induced striatal dopamine losses [60]. *Ccr5*^−/−^ mice showed reduced mDA neurons and increased microglial and astrocytic activation under control conditions [61]. While WT and *Ccr5*^−/−^ mice showed subtle differences in mDA neuron numbers after MPTP administration, the *Ccr5*^−/−^ mice exhibited pronounced dopamine depletion, microglial activation and behavioural deficits as compared to WT mice. Moreover, *Ccr5*^−/−^ mice exhibited an increased immunoreactivity for monoamine oxidase B, higher p38 activation and elevated MPP^+^ levels in the SN and the CPu, indicating the importance of Ccr5 for mDA neuron survival.

Interleukins are a broad class of secreted proteins that mediate various effector functions in response to a stimulus. MPTP treated *Il-1rI*^−/−^ mice showed an aggravated loss of striatal dopaminergic innervation in the CPu and an increased expression of *Igf-1* mRNA, indicating possible interactions between interleukins and neurotrophic factors [62]. *Il-6*^−/−^ mice also showed an increased vulnerability to MPTP in comparison to their wild-type littermates [63]. Striatal dopamine depletion and mDA neuron loss were significantly higher in *Il-6*^−/−^ and *Il-6*^+/−^ mice, which was also dose-dependent. Moreover, striatal astrocytes were immunoreactive for Il-6 after MPTP administration. In a subsequent paper, the same group reported a compromised and transient microglial activation in the SN of *Il-6*^−/−^ mice, 7 days after MPTP administration as compared to WT mice [64]. Astrocytic responses, however, did not differ between the two genotypes. Additionally, iNos expression was localised in both reactive microglia and astrocytes.

Tumour necrosis factor family (Tnf) are a group of pro-inflammatory cytokines predominantly upregulated in serum and tissues following inflammation or infections [65]. Deletion of *Tnf-α* confers protection against MPTP-induced mDA neuron loss, and a reduction of associated neuroinflammatory responses. Using *Tnf-α*^−/−^ mice, Ferger and colleagues showed a complete protection of Th-fibre density in the CPu, with a partial protection against dopamine depletion. However, neither genetic ablation, nor pharmacological inhibition of Tnf-α confer protection in the SN [66].

*Tnf-α*^−/−^ mice also exhibit reduced leakage and better integrity of the BBB after MPTP administration [67]. Morphological changes in microglia were still observed, with reduced activated microglia numbers in *Tnf-α*^−/−^ mice and an attenuated release of Il-1β in the CPu, but normal release in the SN. *Tnf-α*^−/−^ mice also showed an attenuation of microglia and astrocyte numbers after MPTP administration. Interestingly, morphological changes typical for activation were still seen in both cell types [57].

Various groups have investigated the role of Tnf receptors in the context of MPTP-induced neurodegeneration. According to Sriram and colleagues [68,69], double *Tnf-αr1*^−/−^/*Tnf-αr2*^−/−^ mice, but not individual *Tnf-αr1*^−/−^ or *Tnf-αr2*^−/−^ mice, were protected against MPTP-induced mDA neurotoxicity. Increase in MPTP-induced expression of microglial Tnf-α preceded striatal dopaminergic denervation and astrogliosis, which was attenuated in *Tnf-αr1*^−/−^/*Tnf-αr2*^−/−^ mice. On the contrary, Rousselet and colleagues failed to observe protection of mDA neurons in WT, *Tnf-αr1*^−/−^, *Tnf-αr2*^−/−^, or *Tnf-αr1*^−/−^/*Tnf-αr2*^−/−^ double mutant mice after an acute dose of MPTP [70]. However, these *Tnf-αr1*^−/−^/*Tnf-αr2*^−/−^ double mutant mice did show diminished rotarod skills and a decline in striatal dopamine levels. Using a chronic MPTP regime, Leng *et al.* [71] also did not observe differences in striatal dopamine and metabolite levels between WT, *Tnf-αr1*^−/−^ and *Tnf-αr2*^−/−^ mice, underlining the influence of dose and regimes on MPTP-induced degeneration of the nigrostriatal system.

### 2.3. Inflammasome Activation

Inflammasomes are cytosolic multiprotein complexes that are activated upon diverse stimuli, resulting in the production of pro-inflammatory effector cytokines including Il-18 and Il-1β [72,73,74]. These multiprotein complexes form a part of the innate immune response, functioning as sensors and responding to pathogenic stimuli, infections and inflammatory signals [75]. Inflammasomes consist of three components: a cytosolic pattern-recognition receptor, caspase-1, and an adapter protein enabling interaction between them. Upon activation, the cytosolic receptors, including members of the NOD-like receptor (NLR) family (Nucleotide-binding oligomerisation domain receptors), undergo oligomerisation leading to the assembly of the inflammasome complex and a concomitant increase in pro-Il-1β and Nlrp3 levels [72,74]. This serves as a recruitment and activation platform for pro-caspase-1. Activation of caspase-1 leads to the cleavage and release of Il-1β and Il-18, causing inflammatory reactions. Both the ligands and their cognate receptors are expressed in the CNS, where they modulate homeostatic and neuroinflammatory processes [76,77].

Transgenic mice expressing a dominant negative inhibitor of caspase-1 (also known as IL-1β convertase enzyme or ICE), are resistant to MPTP-induced neurotoxicity [78]. As compared to control mice, *caspase-1*^−/−^ mice showed a significant protection against losses of dopamine and its metabolites, as well as of mDA neurons after MPTP administration. The involvement of the Nlrp3 inflammasome in PD has been recently described [79], with functional Nlrp3 inflammasome expression in microglia in response to classical inflammatory activators [80]. Moreover, *Nlrp3*^−/−^ mice are resistant to MPTP-induced degeneration of mDA neurons [81], with reduced circulating levels of Il-1β and Il-18, and an impaired caspase-1 activation. MPTP treatment in type-2 diabetes modelled mice (ob/ob and db/db), led to an impaired insulin signalling, increased mDA neurodegeneration and glial activation in the SN [82]. This was accompanied by elevated Nlrp3 activation and Il-1β release, underling the role of inflammasomes in metabolic mDA neurodegeneration and possibly in PD.

Deletion of the effector cytokines, namely Il-1β and Il-18, also protects against MPTP-induced neuroinflammation and neurodegeneration. *Il-1α/β*^−/−^ mice showed reduced CD68-positive microglia in the CPu and olfactory bulb after MPTP administration [83]. Similarly, *Il-18*^−/−^ mice exhibited reduced mDA loss in the SN [84]. Although microglial activation in the CPu of *Il-18*^−/−^ mice was comparable to that in WT mice, the duration of microglial response was shorter in *Il-18*^−/−^ mice, emphasising the contribution of Il-18 to neuroinflammatory processes. Table 1 summarises the effects of cytokine and chemokine signalling-related gene deletions in the mouse MPTP model.

**Table 1:** Cytokine signalling and inflammasome.

Gene	Gene Description	Receptor	Transgenic Modification	MPTP Administration and Dosage	Neuroinflammation	Neurodegeneration	Reference
Ccl2 (Mcp-1)	Chemokine (C-C motif) ligand 2 chemokine (C-C motif) receptor 2	Ccr2	*Mcp-1/Ccl2* homozygous knockout mice (B6.129S4-Ccl2tm1Rol/J)	4 × 20 mg/kg i.p., 2 h intervals	No differences in RNA expression of *Tnf-α*, *Il-6*, *Cx3cr1*, *Tweak/Tnfsf12*, *Sdf-1/Cxcl12*, *Fn14/Tnfrsf12a* and *Cxcr4.*	No difference in Th-ir mDA neuron numbers	[46]
Ccl2/Ccl3 (Mcp-1/Mip-1α)	Chemokine (C-C motif) ligand 2 Chemokine (C-C motif) receptor 2	Ccr2	Double knockout for CCL2 and CCR2 (*Ccl2^−/−^Ccr2^−/−^*)	3 × 18.2 mg/kg s.c., 2 h intervals	Not described.	Slight reduction in dopamine and DOPAC levels	[60]
Ccl5	Chemokine (C-C motif) receptor 5	Ccr5	*Ccr5^−/−^*	4 × 15 mg/kg i.p., 1.5 h intervals	Microglia activation even under baseline conditions, increased Iba1 protein after MPTP.	Reduced Th-ir mDA neuron numbers and fibre density	[61]
Cx3cl1 (Fractalkine)	Chemokine (C-X-3-C motif) ligand 1 Chemokine (C-X3-C motif) receptor 1	Cx3cr1	*Cx3cl1^−/−^*, *Cx3cr1^−/−^*, *Cx3cr1^+/−^*	4 × 10 mg/kg i.p., 1 h intervals	*Cx3cr1^−/−^* mice exhibit increased microglial activation in the SN after MPTP.	Increased loss of Nissl+ und Th-ir mDA neurons in *Cx3cl1^−/−^*and *Cx3cr1^−/−^* mice as compared to WT mice.	[59]
Cx3cl1 (Fractalkine)	chemokine (C-X-3-C motif) ligand 1 chemokine (C-X3-C motif) receptor 1	Cx3cr1	*Cx3cl1^−/−^*	4 × 10 mg/kg i.p., 1 h intervals	Increased CD68^+^ and CD11b^+^ area in SNpc, increased Tnf-α and ll-1β concentrations in VM.	Reduced Th protein and Th-ir mDA neuron numbers.	[58]
Ifn-γ	Interferon-γ Interferon-γ receptor	Ifn-γr	*Ifn-γ^−/−^*, *Ifn-γr^−/−^*	5 × 25 mg/kg i.p., 24 h intervals	No microglia activation.	Reduced loss of Th-ir mDA neurons and Th protein.	[56]
Il-18 *(Part of the inflammasome)*	Interleukin-18	-	*Il-18^−/−^*	4 × 10 mg/kg i.p., 2 h intervals	Normal microglia activation day 1-3, strongly reduced microglia activation on day 7.	No loss of Th-ir mDA neurons.	[84]
Il-1α	Interleukin-1 α	Il-1r	*Il-1rI^−/−^*	1 × 40 mg/kg s.c.	Higher *Igf-1* mRNA expression.	Reduced DAT on and higher striatal serotonin transporter density.	[62]
Il-1α/β *(Part of the inflammasome)*	Interleukin-1 α Interleukin-1 β	-	*Il-1α/β^−/−^*	4 × 20 mg/kg i.p., 2 h intervals	Decreased percentage of CD68^+^ microglia in olfactory bulb and CPu.	Not described.	[83]
Il-1β *(Part of the inflammasome)*	Interleukin-1 β	-	Dominant negative inhibitor of interleukin-1β convertase enzyme (ICE)	4 × 15 mg/kg, 2 h intervals	Not described.	No reduction in dopamine, DOPAC or HVA after MPTP, no reduction in Th-ir cell count.	[78]
Il-6	Interleukin-6	-	*Il-6^−/−^*	30 mg/kg	No difference in microglial response.	Increased loss of striatal dopamine levels and loss of Th-ir mDA neurons.	[63]
Il-6	Interleukin-6	-	*Il-6*^−/−^	30 mg/kg, s.c.	Normal astrogliosis but compromised microgliosis at day 7; time-dependent decrease in iNos expression.	-	[64]
Nlrp3	NLR family, Pyrin Domain Containing 3	-	*Nlrp3^−/−^*	5 × 20 mg/kg, i.p., 2 h intervals	Reduced Il-1β and Il-18 production; impaired caspase-1 activation.	Resistant to MPTP-induced loss of Th-ir mDA neurons.	[81]
Tnf-α	Tumour necrosis factor-α receptor	Tnf-αr1 and Tnf-αr2	*Tnf-αr1^−/−^*, *Tnf-αr2^−/−^ Tnf-αr1^−/−^Tnf-αr2^−/−^* B6;129S-*Tnf-ar1^tm1 Imx^*, *Tnf-br1^tm1 Imx^*/J	1 × 12.5 mg/kg, s.c.	Attenuated microglial activation in the CPu of *Tnf-αr1^−/−^Tnf-αr2^−/−^* mice.	Reduced Th-ir mDA loss, but exacerbated neuronal damage in hippocampus.	[69]
Tnf-α	Tumour necrosis factor-α	-	*Tnf-a^−/−^*	Acute: 4 × 20 mg/kg i.p., 2 h intervals Sub-acute: 4 × 15 mg/kg i.p., 24 h intervals	Not described.	Reduced losses of striatal dopamine and metabolites, and of striatal Th-ir fibre density; no difference in Th-ir and DA transporter immunoreactivity in the SN; lower mortality (10%).	[66]
Tnf-α	Tumour necrosis factor-α	-	*Tnf-a^−/−^* (B6;129S6-TnftmlGkl/J)	4 × 10 mg/kg i.p., 1 h intervals	Decreased number of activated microglia, reduced pro-inflammatory cytokines (Tnf-α and Il-1β).	Better BBB integrity, no change in Th-ir mDA neuron numbers.	[67]
Tnf-α	Tumour necrosis factor-α receptor	Tnf-αr1 and Tnf-αr2	*Tnf-αr1^−/−^*, *Tnf-αr2^−/−^*, *Tnf-αr1^−/−^Tnf-αr2^−/−^*	1 × 12.5 mg/kg b.w., s.c.	No significant upregulation of Gfap in CPu of *Tnf-αr1^−/−^Tnf-αr2^−/−^* mice.	No striatal Th and dopamine loss in *Tnf-αr1^−/−^Tnf-αr2^−/−^*, still neuronal damage in hippocampus.	[68]
Tnf-α	Tumour necrosis factor α	Tnf-αr1 and Tnf-αr2	*Tnf-αr1^−/−^*, *Tnf-αr2^−/−^*, *Tnf-αr1^−/−^Tnf-αr2^−/−^*	4 × 15 mg/kg b.w., i.p., 2 h intervals.	Not described.	No difference in Th-ir mDA neuron numbers; slight reduction of dopamine in *Tnf-αr1^−/−^Tnf-αr2^−/−^*.	[70]
Tnf-α	Tumour necrosis factor α	Tnf-αr1 and Tnf-αr2	*Tnf-αr1^−/−^*/*Tnf-αr2^−/−^*	8 × 15 mg/kg b.w., i.p., 24 h intervals.	Not described.	No difference in dopamine levels and DAT-ir neurons.	[71]
Tnf-α Ifn-γ	Tumour necrosis factor-α; Interferon-γ		*Ifn-γ^−/−^* (B6.129S7-Ifngtm1Ts/J), *Tnf-α^−/−^* (B6.129S-Tnf. tm1Gkl/J)	1 × 20 mg/kg b.w., i.p.	*Ifn-γ^−/−^*:no microgliosis but morphological change in microglia. *Tnf-α^−/−^*: only slight activation of microglia. *Tnf-α^−/−^*, *Ifn-γ^−/−^*: no astrocyte activation.	No change in Th-ir mDA neuron numbers after 24 h.	[57]

BBB, blood brain barrier; b.w., body weight; DAT, dopamine transporter; DOPAC, 3,4-dihydroxyphenylacetic acid; Fn14, Fibroblast growth factor-inducible 14; HVA, Homovanillic acid; Igf-1, insulin-like growth factor 1; iNos, inducible Nitric oxide synthases; i.p., intraperitoneal injection; Mcp-1, Monocyte chemotactic protein 1; s.c., subcutaneus injection; Sdf-1, Stromal cell-derived factor 1; SNpc, Substantia nigra pars compacta; Th, Tyrosine hydroxylase; Tnfsf-12, Tumour necrosis factor (ligand) superfamily, member 12; Tweak, Tnf-related weak inducer of apoptosis; VM, Ventral midbrain.

## 3. Growth Factors and Neurotrophic Factors

Neurotrophic factors are secreted proteins that play vital roles during development of the nervous system by influencing the maintenance of selective neuronal populations and supporting their survival and maturation through adulthood. Based on these properties, the potential of several neurotrophic factors has been investigated in the parkinsonian brain with the help of animal models and clinical trials [85,86,87]. Here, we provide an update of the neurotrophic factors used in the MPTP mouse model, with a focus on neuroprotection, and the role of glia in these neurodegenerative processes.

Brain-derived neurotrophic factor (Bdnf), a neurotrophin family member, has been shown to promote the survival of mDA neurons of the developing SN, as well as to protect pure mDA neuron cultures from MPP^+^ toxicity [88,89]. Bdnf has also been localised in nestin-positive astrocytes in the CPu of MPTP-lesioned mice, thus highlighting that neurotrophic factors may not only mediate their effects directly on neurons but also through glial cells [90]. Mice heterozygous for Bdnf subjected to 90 days of unrestrictive exercise, are not protected against MPTP-induced neurotoxicity of mDA neurons, suggesting that the full complement of Bdnf is essential for exercise-induced protection of mDA neurons [91]. Signalling via TrkB (Tyrosine receptor kinase B), one of the receptors for neurotrophins including Bdnf, is important for the survival of mDA neurons. Aged *TrkB* hypomorphic mice expressing the receptor at a quarter to a third of the normal amount show a significant loss of mDA neurons in the SN accompanied by an increase in reactive gliosis. Furthermore, the *TrkB* hypomorphic mice exhibit a significantly greater reduction in the number of mDA neurons after MPTP administration, indicating that a reduction in TrkB signalling increases the vulnerability of these neurons to neurotoxin-induced cell death [92]. Exogenous application of nerve growth factor (Ngf), another member of this family, has been shown to be beneficial for the nigrostriatal system by increasing the dopamine content in the CPu of MPTP-lesioned mice [93]. Moreover, Ngf and neurotrophin-3 were found to co-localise in reactive astrocytes in the CPu of MPTP-lesioned mice [94], underlining the neuroprotective roles of reactive astrocytes in PD.

The neuroprotective and neurorestorative potentials of several members of the Transforming growth factor-β (TGF-β) superfamily have been investigated in the MPTP neurotoxic model [95]. Of these, Glial derived neurotrophic factor (Gdnf) has received the most attention as a potential therapeutic agent for treating PD [96,97]. Mice with null mutations for *Gdnf* die at birth making it impractical to assess its post-natal effects on the nigrostriatal system. Granholm and colleagues circumvented this by transplanting foetal neural tissues from *Gdnf*^−/−^, *Gdnf*^+/−^ and WT mice into the CPu of MPTP-treated adult WT mice [98]. Grafts from *Gdnf*^−/−^ foetal ventral midbrains have reduced mDA neuron numbers and fibre outgrowth. Moreover, these grafts can be rescued by pre-incubation with Gdnf before grafting, highlighting its neurorestorative effects in this model. Exogenous application of Gdnf either via stereotactic injections [99,100,101], via gelfoam application [102], or via a lentiviral construct [103], have shown to be neuroprotective for the MPTP-lesioned nigrostriatal system. Of note, lentiviral mediated expression of *Gdnf* under a macrophage specific promoter, ameliorated MPTP-induced neurodegeneration in recipient mice, via putative neuroprotective effects of Gdnf expressing macrophages or microglia on mDA neurons [103]. Similarly, bone marrow-derived microglial delivery of Neurturin (Ntn), a member of the Gdnf family of ligands, also led to a significant protection of mDA neurons and their fibre terminals post MPTP administration [104]. Conditional deletion of the Gdnf receptor *Ret* (Rearranged during transfection) did not increase the vulnerability of mDA neurons after MPTP lesion, but was essential for the reappearance of fibres in the CPu [105]. Moreover, MPTP treated mice haploinsufficient for the Gdnf receptor *Gfrα1* (Glial cell line derived neurotrophic factor family receptor α 1), showed increased death of mDA neurons accompanied by an elevated inflammatory response consisting of reactive microglia and CD45-ir in the SN [106]. Given the importance of Tgf-βs in the developing nigrostriatal system [107] and its requirement to elicit neuroprotective effects in conjunction with Gdnf [108,109], Schober and colleagues simultaneously applied exogenous Gdnf in combination with Tgf-β neutralising antibodies to an MPTP lesioned nigrostriatal system [102]. The neuroprotective effects of Gdnf on MPTP-lesioned mDA neurons and their terminal fibres were abolished in the absence of Tgf-β. This emphasises the requirement of Tgf-β as an essential co-trophic factor for Gdnf in the MPTP mouse model of PD. Mice haploinsufficient for *Tgf-β2* however do not show a reduction in MPTP-induced striatal dopamine depletion [110]. On the contrary, over expression of *Tgf-β1* in the nigrostriatal system of MPTP lesioned mice caused significant reduction in the numbers of mDA neurons and an extensive depletion of dopamine levels in the CPu [111].

The potential of gliotrophic factors such as the Fibroblast growth factor (Fgf), Epidermal growth factor (Egf) and Insulin-like growth factor (Igf) have also been investigated in the MPTP mouse model of PD. Intracerebroventricular infusion of Egf partially enhanced striatal dopamine levels and further increased the activity of Th in MPTP treated mice, suggesting a neurotrophic role for mDA neurons [112]. Fgf can induce glial proliferation *in vivo* and *in vitro*, and is upregulated following brain injury as an essential molecule for scar formation [97]. Stereotactic injections of Fgf-1 (also known as acidic Fgf) into the CPu of MPTP-lesioned young and old mice, led to a recovery of dopamine concentrations and striatal fibre densities in young but not in old mice, indicating that the benefits of exogenous application declined with increasing age [113]. Exogenous application of Fgf-2 via a striatal gelfoam implantation, or administration of human recombinant Fgf in MPTP treated mice, ameliorated toxin-induced degeneration the nigrostriatal system [114,115,116]. Although Fgf-2 application did not lead to a sustained increase in astroglial numbers [117], Fgf-2-ir was observed in both astroglial and microglial cells post MPTP administration, implying the neuroprotective functions of glial cells in this paradigm [118]. However, in spite of its prominent role as a neurotrophic factor for mDA neurons, MPTP lesioned *Fgf-2*^−/−^ mice do not exhibit differences in dopamine levels or mDA neuron numbers as compared to lesioned WT littermates [119]. This may be partly attributed to compensatory effects by another member of the Fgf family, or by another growth factor family altogether.

Igfs are essential trophic factors required for the growth and development of the embryonic brain, with neuromodulatory functions in the adult brain [97,120]. The protein encoded by the gene *Igf-1* is served by its receptor Igf-1r, a transmembrane tyrosine-kinase receptor homologous to the insulin receptor. Mice haploinsufficient for the *Igf-1r* administered with MPTP show exacerbated losses of mDA neurons as compared to their WT littermates. Moreover, the *Igf-1r^+/−^* mice exhibit an increased microglial inflammatory response to the neurotoxin and a down-regulation of several anti-inflammatory pathways under control conditions, indicating that the Igf signalling pathway/s can reduce neuroinflammatory responses and sensitivity of mDA neurons to MPTP-induced inflammation [121].

Members of the Platelet derived growth factor (Pgdf) family also act via tyrosine kinase receptors and regulate cellular proliferation, growth and differentiation [97,122]. The Pdgf-bb isoform has been shown to increase the survival of cultured mDA neurons and induce neurite outgrowth [123,124]. Intracerebroventricular administration of Pgdf-bb following MPTP lesion, restored striatal dopamine transporter binding sites and Th expression, and led to an increase in cell proliferation in the CPu of lesioned mice [122]. The ability of Colony stimulating factors (Csfs), namely granulocyte Csf (G-Csf) and Granulocyte-Macrophage Csf (GM-Csf), has recently been realised in the diseased CNS. While G-Csf specifically promotes neutrophil proliferation and maturation, GM-Csf has effects on multiple cell lineages including macrophages and eosinophils [125]. G-Csf is commonly used to treat neutropenia, and has been shown to have roles in the challenged nervous system [126,127]. Exogenous administration of G-Csf in MPTP treated mice rescued mDA neuron death and recovered striatal dopamine levels [128,129]. Moreover, this neuroprotective effect could be elicited by modulation of Bcl-2 and Bax expression in the injured nigrostriatal system [130]. MPTP-lesioned mice post-treated with G-Csf showed a reduced microglial burden in the CPu, indicating a putative neuroprotective and restorative effect [129]. In the CNS, GM-Csf is produced by resident astroglial cells, fibroblasts, endothelial cells and cells of the myeloid lineage [125]. MPTP-lesioned mice receiving exogenous GM-Csf showed reduced mDA loss, increase in dopamine levels, and an improved motor performance [131], with reductions in microglial activation and responses, and an induction of regulatory T-cells suggestive of the neuroprotective effects of GM-Csf [132].

The recently discovered Cerebral dopamine neurotrophic factor (Cdnf) is a member of the conserved Cdnf/Manf family of neurotrophic factors, and has been shown to protect and rescue 6-OHDA-lesioned mDA neurons *in vivo* [133,134]. As in the MPTP model, Cdnf administered before or after MPTP lesion led to an increase in striatal Th-fibre densities and increased neuron survival, with improved locomotor behaviour, thus underscoring its neuroprotective and neurorestorative effects on the injured nigrostriatal system [135]. A comprehensive overview of growth factors and neurotrophic factors is given in Table 2.

## 4. Concluding Remarks

**Figure 1:**
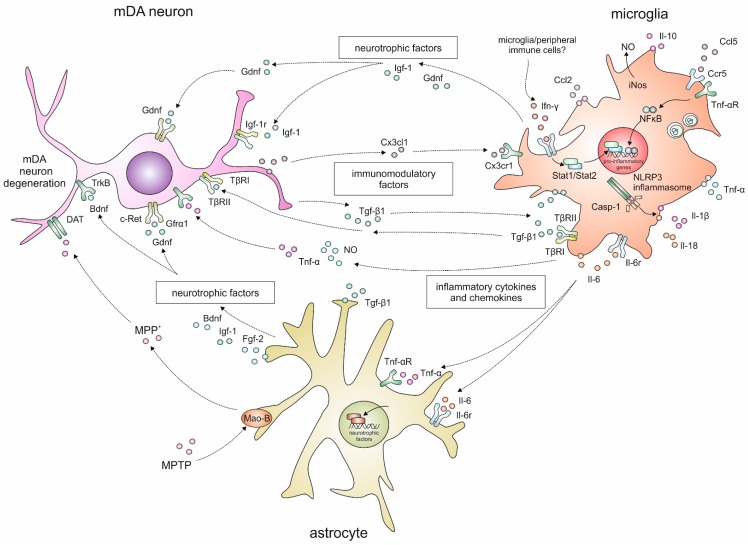
Schematic overview of the complex interactions between mDA neurons, microglia and astroglia after MPTP applications in mice.

In the present review, we have highlighted the involvement of microglia-mediated neuroinflammation and effects of neurotrophic factors in the mouse MPTP model of PD. Figure 1 summarises the complex interactions between neurons, microglia and astrocytes. The studies performed with respective transgenic mice demonstrate that microglia reactions and the subsequent release of cytokines contribute to an increased degeneration of mDA neurons after MPTP administration. However, most of the studies showed insufficient protection of mDA neurons when cytokines or their respective receptors had been silenced, indicating that MPTP-driven neurotoxicity is enhanced, but not caused by neuroinflammation. Neurotrophic factors are, notwithstanding initial failed attempts, the most promising therapeutic option to slow progression of mDA neuron loss in PD patients. Interestingly, studies using either exogenous application of neurotrophic factors or transgenic mice, with factor and/or receptor deletions, support the potential of neurotrophic factors as protective cues in the mouse MPTP model. Thus, we propose that future therapeutic strategies should consider inhibition of neuroinflammation and direct neuroprotection as two separate phenomena, which can be modified therapeutically. Based on the current data, combinations of potent neurotrophic factors and inhibitors, or modulators of microglial reactions, might be appropriate and promising to slow the progression of PD.

**Table 2:** Growth factors and neurotrophic factors.

Gene	Gene Description	Transgenic Mouse/Exogenous Treatment	Transgenic Modification	MPTP Administration and Dosage		Neuroinflammation	Neurodegeneration	**Reference**
Bdnf	Brain-derived neurotrophic factor	*Bdnf*^+/−^ mice	Haploinsufficient	Yes, 4 × 20 mg/kg b.w., i.p. at 2 h intervals		Not described.	*Bdnf*^+/−^ mice do not show differences compared to WT mice.	[91]
Cdnf	Cerebral dopamine neurotrophic factor	Pre and post treatment with Cdnf 5 μg/μL (bilateral striatal injections) before and after MPTP	None	Yes, 4 × 15 mg/kg b.w, i.p. for pre-treatment and 20mg/kg b.w, i.p. for post-treatment respectively		Not described.	Exogenous Cdnf proves neuroprotective and neurorestorative for the NS system.	[135]
Egf	Epidermal growth factor	Infusion of Egf 5 μg/week	None	Yes, 7 × 30 mg/kg b.w., i.p.		Not described.	Partial restoration of dopamine and DOPAC.	[112]
aFgf or Fgf-1	acidic Fgf or Fibroblast growth factor-1	Stereotactic injections of aFgf 0.5 μg/μL	None	Yes, 4 × 20 mg/kg b.w., i.p.		Not described.	Dopamine concentration and striatal fibre density partially recovered in young mice (8 w) but not in old mice (12 m).	[113]
bFgf or Fgf-2	Fibroblast growth factor-2	*Fgf-2*^−/−^	Complete knockout	Yes, 3 × 20 mg/kg b.w., i.p.		Not described.	No significant differences in the NS system between WT and *Fgf-2*^−/−^ mice.	[119]
Fgf-2	Fibroblast growth factor-2	Gelfoam containing 4 μg Fgf-2 or cytochrome-c	None	Yes, 3 × 20 mg/kg b.w., i.p.		Co-staining of Fgf-2 with presumed microglial cells.	Not described.	[118]
Fgf-2	Fibroblast growth factor-2	Gel foam containing 4 μg Fgf-2 or cytochrome-c	None	Yes, 3 × 20 mg/kg b.w., i.p.		Not described.	Moderates reduction of striatal dopamine and reverses losses of Th-ir mDA neurons.	[114]
Fgf-2	Fibroblast growth factor-2	Gel foam containing 4 μg Fgf-2 or cytochrome-c	None	Yes, 3 × 20 mg/kg b.w., i.p.		No excessive reactive astrocytosis after Fgf-2 application.	Striatal dopamine content was checked to asses MPTP effect.	[117]
Fgf-2	Fibroblast growth factor-2	Intraventricular infusion of hrFGF-22 μg/24 h	None	Yes, 40 mg/kg b.w., s.c		hrFGF-2 induces increase in astroglial reaction and numbers in SN and CPu.	hrFGF-2 treatment reduced MPTP-induced losses of mDA neurons and fibres. Locomotor activity was fully recovered after hrFGF-2 treatment.	[115]
G-Csf	Granulocyte-colony stimulating factor	8x250 μg/kg, b.w., s.c.	None	Yes, 5 × 30 mg/kg b.w., i.p.	G-csf treatment after MPTP reduced microglial burden in the CPu.		G-csf treatment after MPTP improved rotarod performance.	[129]
Gdnf	Glial derived neurotrophic factor	Transplants of foetal neural tissues (*Gdnf*^−/−^ or *Gdnf*^-/+^ or *Gdnf*^+/−^) into MPTP treated adult WT mice	Gdnf^−/−^ or Gdnf^+/−^ foetal neural tissues transplanted into ventral CPu of MPTP lesioned WT mice	Yes, 4 × 30 mg/kg/day	Not described.		*Gdnf*^−/−^ grafts pre-incubated with Gdnf show increased Th-ir mDA neuron numbers.	[98]
Gdnf	Glial derived neurotrophic factor	Unilateral stereotactic injections of Gdnf 5 g/μL	None	Yes, 4 × 20 mg/kg b.w., i.p.	Not described.		Gdnf administration induces recovery of the NS system in both young and aged mice.	[99]
Gdnf	Glial derived neurotrophic factor	Intracerebral injections of Gdnf (5 μg/μL) before and after MPTP	None	Yes, 2 × 40 mg/kg b.w., s.c.	Not described.		Protection of mDA neurons; recovery of Th fibres and dopamine in the CPu. Motor behaviour increased above normal levels.	[100]
Gdnf	Glial derived neurotrophic factor	Intrastriatal injections of Gdnf 2 μg/μL	None	Yes, 7 × 35 mg/kg, b.w., s.c.	Not described.		Increased locomotor activity, striatal dopamine and metabolite levels, Increase in Th-ir mDA neurons.	[101]
Gdnf	Glial derived neurotrophic factor	Gdnf lentiviral construct in a macrophage-specific synthetic promoter	None	Yes,15 mg/kg b.w., free base MPTP on day 1, 25 mg/kg b.w., on day 2, and 30 mg/kg b.w., on days 3–7, s.c.	Putative neuroprotective effects of Gdnf expressing macrophage/microglia on Th-ir mDA neurons.		Macrophage-mediated Gdnf treatment ameliorated MPTP-induced degeneration of mDA neurons and Th fibre terminals, stimulated axon regeneration, and reversed hypoactivity in the open field test.	[103]
Gdnf	Glial derived neurotrophic factor	Gel foam containing 1 μg/μL Gdnf or cytochrome-c	None	Yes, 3 × 20 mg/kg b.w., i.p.		Not described.	Exogenous Gdnf reduced loss of striatal DA fibres.	[102]
Gfrα1	Gdnf receptor (Glial cell line derived neurotrophic factor family receptor α 1)	*Gfrα1*^+/−^	Haploinsufficient; substitution with phosphoglycerate kinase	Yes, 4 × 20 mg/kg b.w., i.p.		Higher CD45-ir, microglial response in the SN.	Increase in Th-ir mDA neuron death.	[106]
Gm-Csf	Granulocyte-macrophage-colony stimulating factor	5x50 μg/kg i.p.	None	Yes, 4 × 16 mg/kg b.w., s.c.		Gm-csf pre-treatment altered microglial morphology (reduced microgliosis) and Treg induction.	Neuroprotection of Th-ir mDA neurons and striatal fibres by adoptive transfer of Gm-Csf-induced Treg to MPTP mice.	[132]
Igf-1r	Insulin-like growth factor-1 receptor	*Igf-1r*^+/−^	Haploinsufficient	Yes, 2 × 30 mg/kg b.w., i.p. at 3 h intervals		Increase in neuroinflammatory responses (particularly microglia numbers in SNpc and SNr).	Increase in Th-ir mDA neuron death.	[121]
Ngf	Nerve growth factor	Stereotactic injections of 0.4 μg/μL Ngf into the right ventricle	None	Yes, 5 × 30 mg/kg b.w., i.p.		Not described.	Restoration of dopamine and HVA levels after Ngf injections.	[93]
Ntn	Neurturin	Ntn lentiviral construct in a microglia specific synthetic promoter	None	Yes, 15 mg/kg b.w., free base MPTP on day 1, 25 mg/kg b.w., on day 2, and 30 mg/kg b.w., on days 3–7, s.c.		Neuroprotective effects of Ntn expressing microglia on mDA neurons.	Reduction in MPTP-induced degeneration of mDA neurons in the SN and fibre terminals in the CPu.	[104]
Pdgf	Platelet derived growth factor	Pdgf-bb delivery (36 ng/day) for 2 w into the right lateral ventricle via osmotic pumps	None	For cell proliferation: 4 × 15 mg/kg b.w., i.p. For neurorestoration 1 × 40 mg/kg b.w., s.c.		Not described.	Pgdf-bb administration lead to an increase in striatal Th expression and DAT sites.	[122]
Ret	Gdnf receptor (rearranged during transfection)	*Dat-Ret*^lx/lx^	Conditional knockout	Yes, 5 × 30 mg/kg b.w., i.p.		No difference in MPTP-induced astrogliosis in CPu.	Ret deficiency does not increase MPTP vulnerability in the SN, but is essential for regeneration in the CPu.	[105]
Tgf-β2	Transforming growth factor-β 2	*Tgf- β*2^+/−^	Haploinsufficient	Yes, 40 + 20 mg/kg b.w., s.c.		Not described.	Marginally reduced Th-ir mDA neurons. Reduced striatal dopamine turnover.	[110]
Tgf-β	Transforming growth factor-β	Gel foam containing anti Tgf-β pan mAB or isotype control mouse IgG (5 μg)	None	Yes, 3 × 20 mg/kg b.w., i.p.		Not described.	Simultaneous application of Gdnf and Tgf-β neutralising antibodies abolished the neuroprotective effects of Gdnf.	[102]
Tgf-β1	Transforming growth factor-β 1	AAV- mediated *Tgf-β1* overexpression	None	Yes, 30 mg/kg b.w., s.c.		Not described.	Overexpression of Tgf-β1 in the NS aggravates the Parkinsonian state caused by MPTP injury in adult mice.	[111]
TrkB	Bdnf receptor (Tyrosine receptor kinase B)	TrkB hypomorphic mutant, fBneo/fBneo (expresses *TrkB* at 1/4-1/3 of the normal amount)	Conditional knockout	Yes, 5 × 25 mg/kg b.w., i.p.		Increased reactive astrogliosis in the CPu of mutant mice.	Mutant mice exhibit selective and late neurodegeneration. mDA neurons show enhanced vulnerability to MPTP.	[92]

AAV, Adeno-associated virus; b.w., body weight; CD45, Cluster of Differentiation 45; CPu, Caudato putamen; DAT, Dopamine transporter; DOPAC, 3,4-Dihydroxyphenylacetic acid; hrFgf-2, Human recombinant Fgf-2; HVA, Homovanillic acid; i.p., Intraperitoneal; mAB, Monoclonal antibody; NS, Nigrostriatal; s.c., subcutaneous; SN, Substantia nigra; SNpc, Substantia nigra pars compacta; SNr, Substantia nigra pars reticularis; Treg, Regulatory T-cell.
